# Acoustic Emission-Based Study to Characterize the Crack Initiation Point of Wood Fiber/HDPE Composites

**DOI:** 10.3390/polym11040701

**Published:** 2019-04-17

**Authors:** Yong Guo, Shiliu Zhu, Yuxia Chen, Dian Liu, Dagang Li

**Affiliations:** 1 School of Forestry and Landscape Architecture, Anhui Agricultural University, Hefei 230036, China; zhuslwood@163.com (S.Z.); sheherose@163.com (Y.C.); 18955967695@163.com (D.L.); 2 College of Materials Science and Engineering, Nanjing Forestry University, Nanjing 210037, China

**Keywords:** wood fibers, fracture toughness, damage mechanics, acoustic emission

## Abstract

The crack initiation point can be regarded as a sign of composite failure and plays a vital role in the evaluation of fracture toughness. Wood-plastic composites (WPCs) are viscoelastic materials and the evaluation of fracture mechanism and toughness has a great significance in their applications. Therefore, we used the acoustic emission (AE) technique to measure the crack initiation point of the WPCs and evaluate their fracture toughness. The results show that the novel AE-based methods were more effective than the conventional standard methods for characterization of the crack initiation point. Using the relationship of cumulative AE events with time and load, the critical failure load was quickly determined, and then the critical stress intensity factor and fracture toughness were calculated. The fracture toughness of the WPCs increased with an increase in the wood fiber content.

## 1. Introduction

Fracture toughness is an inherent property of a material which depends on its composition and structure and can be employed to characterize the ability of a material to resist crack propagation [1,2]. The fracture behavior of polymer composites consists of processes starting from microscopic damage to crack initiation and propagation, ultimately leading to complete failure. The aim of this research is to investigate the conditions and regularity of crack initiation and propagation. In general, the preferred structure can be obtained by adjustment of the raw material (including the additives like coupling agent), formulation design, molding, and processing techniques, leading to enhancement of the fracture toughness of the polymer composites and improvement of the bearing capacity of the cracked structural components.

The study of crack propagation in fracture mechanics primarily includes stress analysis and energy analysis methods. Stress analysis highlights that the critical state of crack propagation is determined by the critical stress intensity factor (*K_I_*), while the energy analysis emphasizes the critical strain energy release rate (*G_Ic_*) when the crack propagates [1]. These are primarily used as the fracture failure criteria for linear elastic fracture mechanics. However, the wood-plastic composites (WPCs) are nonlinear viscoelastic materials [3,4], and still, no uniform standards exist for testing their fracture toughness. In general, it is assumed that the plastic region of the crack tip in WPCs is extremely small, and hence, studied via stress analysis using linear elastic fracture mechanics. However, the results obtained using the two approaches are significantly different, and there is a lack of comparative study between the related methods.

As a reliable, non-destructive testing technology, acoustic emission (AE) has emerged with a wide range of applications in the field of damage detection and quality evaluation of materials. The technology can monitor and identify stress wave signals of the composites ranging from microscopic deformation to the fracture process, including the crack initiation, propagation, delamination, and fracture [5,6,7,8,9,10,11,12,13]. The AE activity and AE intensity can be utilized to characterize the kinetics of the delamination process in Mode I fracture toughness tests for fiber-reinforced polymer laminates and provide reliable information on the delamination onset at both the microscopic and macroscopic scales [10,14]. The different damages in laminated composites can also be efficiently distinguished [11], and delamination propagation can be identified earlier [15]. In addition, the AE parameters and logarithm of the ratio between the mechanical and acoustic energies can aid in detection of the initiation and growth of delamination as well as in evaluation of the delamination fracture toughness in the composite laminates [16,17,18,19]. Furthermore, low interlaminar strength and the presence of damage/flaws in the material can be identified via the combination of the AE technique and wavelet analysis [20]. The fiber breakage, fiber stripping from the substrate or spalling, matrix cracking, and interfacial can be evaluated and analyzed using AE signals including the amplitude, count, rise time, duration, and energy [21,22,23,24,25]. The crack tip location during the fracture process can be identified using the AE signal source localization and cumulative AE energy [19,26,27]. The AE technique has unique advantages for studying the initiation and propagation of cracks and can better describe the damage and fracture behavior of materials along with evaluating fracture toughness. However, the majority of these studies have focused on the glass fiber- or carbon fiber-reinforced polymer laminate composites, and there are limited studies on the WPCs, especially the three-point bending mode.

Hence, we used AE technology to evaluate the fracture toughness of the WPCs under the three-point bending test and compared them with the traditional stress intensity factor analysis (standard method). Specifically, the determination of the crack initiation point and the calculation of *K_I_* have a vital role in the fracture analyses of the structures. Understanding initial crack creation and propagation is a particular concern for the WPCs in the field of furniture and construction, because the damage processes of the composite are crucial for long-term applications [28]. The results of this work may provide theoretical support for a better design and use of WPCs and expand and enrich the quality performance evaluation method of the WPCs. 

## 2. Materials and Methods

### 2.1. Materials

**Figure 1:**
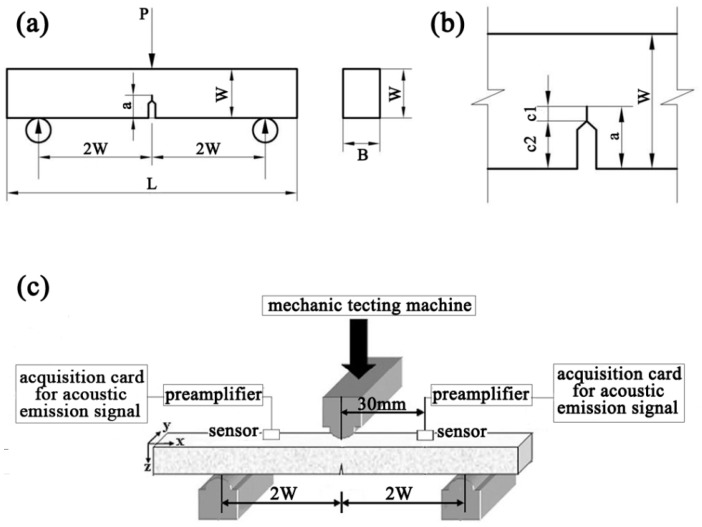
The specimen for the three-point bending test ((**a**) the specimen; (**b**) details of the notch-crack) and the schematic of the experimental device (**c**); (S = 200 mm; B = 20 mm; W = 30 mm; a/W = 0.5).

The materials used in this work were wood fiber/recycled high-density polyethylene (WF/Re-HDPE) composites with 50%, 60%, and 70% wood fiber (weight percent (wt %), recorded as 50% WF/Re-HDPE, 60% WF/Re-HDPE, and 70% WF/Re-HDPE, respectively). The specific formulation and preparation process can be found in our previous work [7]. The prepared composite sheet was processed into a single-edge-notch bending (SENB) toughness test piece using a precision push bench saw (CS70EB, Festool, Wendlingen, Germany). The dimensions are shown in Figure 1. The portion of length c1 was sawn with a hacksaw blade and the portion of length c2 was sharpened with a blade. However, the front edge was not extremely straight and prefabrication of the crack front was done with a sharp blade. Therefore, after the sample was broken, the fractured sample was taken, and depth a1, a2, and a3 of the crack at 1/4, 1/2, and 3/4 of the width of the sample, respectively, were measured, and finally, the arithmetic mean was taken as the effective crack length (a).

### 2.2. Characterization

The critical stress intensity factor (*K_I_*) test for WPCs was performed in accordance with ASTM E 399-2012 [29] and Chinese standard GB/T 4161-2007 [30]. The specimen was pressurized in the three-point bending mode using a universal testing machine (AUTOGRAPH AG-IC, Shimadzu, Kyoto, Japan) (see Figure 1). The span and the loading speed were 120 mm and 2 mm/min, respectively. The load and displacement during the test were automatically recorded using the load sensor and the displacement sensor (an electrical output double-cantilever clip-in displacement gage) and automatically provided the P–V curve for the relationship between the load (P) and the crack tip opening displacement (V). However, at the time of cracking, *K_I_* primarily depends on the critical load when the crack appears; hence, the determination of load at the crack initiation point is particularly crucial. To effectively determine the crack initiation point load, the AE signal acquisition system (PCI-2, Physical Acoustics Corporation (PAC), Princeton, NJ, USA) was employed to collect the AE signals produced by the stressed specimen; the test device is shown in Figure 1c. The sensor receives the AE signal, transmits it to the preamplifier, and then, collects and records it through the computer equipped with the AE acquisition card. The laboratory remained quiet and noise free at the test temperature of 25 °C, and a relative humidity of 35–40% was maintained throughout the experiment.

The calculation of the *K_I_* was performed according to formula (1) [29,30], where $f(\frac{a}{W})$ is the correction factor for the SENB specimen. $f(\frac{a}{W})$ was calculated as per formula (2) [29,30],

(1) $$K_{I}=\frac{P_{Q}S}{BW^{3/2}}f(\frac{a}{W})$$

(2) $$f(\frac{a}{W})=2.9{\left(\frac{a}{W}\right)}^{1/2}-4.6{\left(\frac{a}{W}\right)}^{3/2}+21.8{\left(\frac{a}{W}\right)}^{5/2}-37.6{\left(\frac{a}{W}\right)}^{7/2}+38.7{\left(\frac{a}{W}\right)}^{9/2}$$

where *P_Q_* is the critical failure load, S is the span, B is the specimen thickness, W is the specimen width, and a is the crack length. The values of a/W range from 0.45 to 0.55. If the specific value of *P_m_*/*P_Q_* (where *P_m_* is the maximum load, N) is lower than 1.1 [29,30], the results are valid. *K_I_* is the plane strain fracture toughness (*K_Ic_*) of the material and *K_I_* = *K_Ic_*. 

## 3. Results

### 3.1. Fracture Toughness of High-Filled WPCs Determined Using Conventional Standard Method

**Figure 2:**
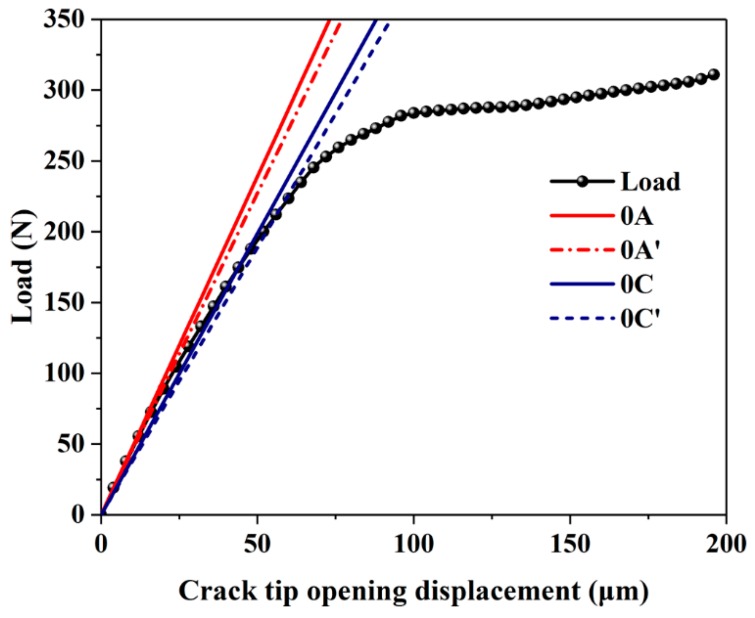
P–V curve of the relationship between the load (P) and the crack tip opening displacement (V) (0A, 0C—the tangent of the selected initial linear portion, and 0A’, 0C’—the secant lines where the slope is 95% of the slopes of 0A and 0C.)

The key to calculating *K_Ic_* is to determine the value of *P_Q_*. The P–V curve of the 50% WF/Re-HDPE composites under the three-point bending test is shown in Figure 2. The lines of 0A and 0C are the tangent of the selected initial linear portion, while 0A’ and 0C’ are the secant lines where the slope is 95% of the slopes of 0A and 0C. In this experiment, the best fitting line can be obtained by fitting the approximate initial linear stage with origin 9.1 software. Then, the origin 9.1 software can be used to draw the tangent to get 0A or 0C. In addition, according to the ASTM standard ASTM E 399-2012 and Chinese standard GB/T 4161-2007, for Type I load–displacement curves (the WPC’s P–V curve in this experiments was closer to Type I), the force *P_Q_* is defined as follows: if the force at every point on the record which precedes *P_S_* is lower than *P_S_* (*P_S_* is the load corresponding to the intersection of the secant and the P–V curve), then *P_S_* is *P_Q_*. Therefore, the load corresponding to the intersection of the secant and the P–V curve is *P_Q_*. To calculate the fracture toughness using the standard method, the *K_I_* was calculated according to the formula (1) and the formula (2) by selecting a relatively reasonable critical load *P_Q_*, and the validity was verified by the specific value of *P_m_*/*P_Q_* and, thereafter, the *K_Ic_* was determined. 

Table 1 provides the slopes of different test points on the P–V curve (see Figure 2) and the corresponding critical load. The arbitrary nature of the linear slope selection seriously affected the values of the critical load and, thus, the fracture toughness of the high-filled WPCs could not be accurately calculated. Also, the ratio of the maximum load to the critical load (*P_m_*/*P_Q_*) was greater than 1.1; therefore, this experiment is not a valid *K_Ic_* experiment [29,30]. These results indicate that the use of this method to determine the critical load has certain limitations, which is primarily because it is an empirical method that was established after a large number of experiments on metals. However, the WPCs are viscoelastic materials and the P–V curve is basically nonlinear [3,4]. It is extremely challenging to select a straight line that can characterize the linear characteristics of the WPCs. Therefore, the determination of *P_Q_* using the said method has certain subjectivity, and the results have certain errors. The obtained fracture toughness may not truly reflect the inherent properties of the WPCs. 

### 3.2. Fracture Toughness of High-Filled WPCs as Determined Using AE Technology

**Figure 3:**
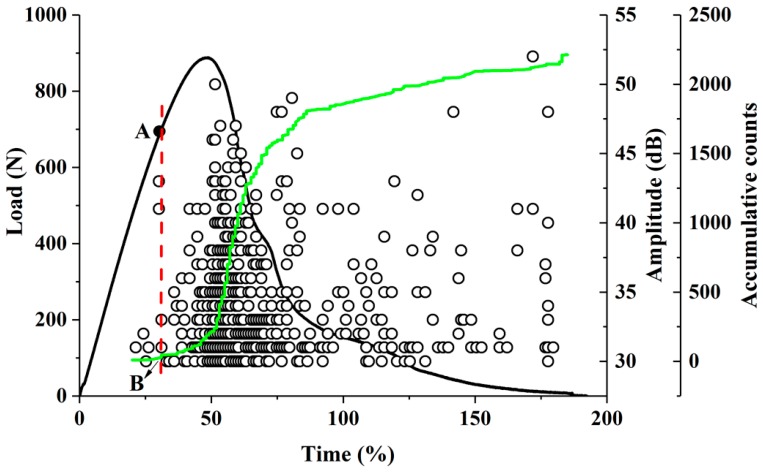
Acoustic emission (AE) amplitude and accumulative counts varied with load-time during the three-point bending test. The black curve represents the load–time curve, the green curve represents the AE accumulative counts, and the black circle represents the amplitude; point A is the intersection of the vertical line (red dotted line) and the load–time curve over point B represents the crack initiation point; point B is the point at which the slope of the accumulative counts curve first mutates.

AE provides a novel method for the determination of the critical load due to its unique sensitivity to defects, damage initiation and expansion, and the initiative and dynamics of detection. Our previous work has shown that the damage of the high-filled WPCs has three stages, namely, the first stage is matrix deformation (elastic deformation), the second stage is fiber breakage and debonding (crack initiation and propagation), and the third stage is increased fiber debonding and fiber pull out, until matrix cracking to fracture (breaking stage) [7]. Therefore, fiber breakage and debonding are the signs of crack initiation and propagation. We defined the moment when the fiber breakage or debonding appeared for the first time as the crack initiation point. In this experiment, as the loading continues, the slope of the accumulative counts curve (the green curve in Figure 3) slowly increased, and the first sudden increase occurs when cracks were generated. Thereafter, the slope continues to increase as the crack propagates until a second burst occurs when the sample fractured. The crack initiation point was obtained according to the first mutation starting point (point B in Figure 3) of the slope at the curve of the AE accumulative counts versus time. The load corresponding to the crack initiation point was referred to as *P_Q_* (the load at point A in Figure 3) and could be directly employed to calculate the *K_Ic_* of the high-filled WPCs. 

Table 2 shows the values of *P_Q_* and *K_Ic_* for the high-filled WPCs with different wood fiber contents as determined using the standard method and AE technique. These results suggest that the *K_Ic_* calculated using AE completely satisfies the requirements of the value of *P_m_*/*P_Q_* lower than 1.1. Therefore, the calculation results were reasonable, and *K_Ic_* is the plane strain fracture toughness of the WPCs. By comparison of the two methods, *K_Ic_* obtained using AE to determine *P_Q_* was greater than the *K_Ic_* obtained by obtaining the *P_Q_* from the P–V curve. This is primarily because the standard method for testing the fracture toughness mainly applies to metals. The stress–strain curve and P–V curve of the metallic materials have an extremely good linear relationship. However, the WPCs are viscoelastic [3,4], and the stress–strain curve and P–V curve are all nonlinear. Furthermore, as shown in Figure 2 and Table 1, the slope of the P–V curve constantly decreased, resulting in a gradual decrease in the corresponding critical load as the measuring point was further away from the origin. However, AE signal does not utilize the P–V curve and is not affected by its nonlinearity. These results suggest that the use of AE technology to determine the critical load and calculate the fracture toughness has a clear physical meaning which can reflect the true mechanical properties and fracture resistance of the WPCs. The value of the stress intensity factor is basically similar to the literature studies on PP/wood composites [31] and wood-flake reinforced polyester composites [32], while it differs from Agathis (*Agathis* sp.) lumber [33,34] and medium-density fiberboard [35]. It is an intuitive and accurate method for testing fracture toughness. 

Moreover, the fracture toughness of the high-filled WPCs with different wood fiber contents as calculated by AE technology are shown in Table 3. The fracture toughness of WPCs increased with the increasing wood fiber content, and the variable coefficient of the three groups of experiments was small. These results indicate that the fiber-supporting effect of the fiber in the WPCs was enhanced with the increasing wood fiber content, and the ability to resist fracture increased.

## 4. Conclusions

This paper used the AE technique to analyze the fracture toughness of the high-filled WPCs. The results were compared with those measured as per the standard methods. In addition, the results show that the AE technique has great potential in the study of damage and fracture characteristics of the WPCs. The crack initiation point of the WPCs could be quickly and accurately determined based on the cumulative AE events. Then, the critical failure load was determined as per the corresponding relationship between the cumulative AE events and the time and load. Also, the critical stress intensity factor was calculated and the fracture toughness of WPCs was determined. These results are more realistic and accurate compared to the ones obtained using the standard method. In addition, the study showed that the addition of wood fibers increases the fracture toughness of the WF/HDPE composites. The fracture toughness of the wood fiber/HDPE composites was increased by 33.33% as the wood fiber content increased from 50% to 70%. 

## Figures and Tables

**Table 1:** Critical loads at different test points as determined from the P–V curve.

Test Point	Coordinates of the Point	k (N/μm)	k_0.95_ (N/μm)	*P_Q_* (N)	*P_m_* (N)	*P_m_*/*P_Q_*
1	(0.0, 0.0)	-	-	-	-	-
2	(1.5, 5.9)	4.780	4.541	67.1723	581.157	8.65
3	(3.0, 12.0)	4.719	4.483	78.599	581.157	7.39
4	(4.4, 18.6)	4.626	4.394	96.713	581.157	6.01
5	(7.4, 32.9)	4.532	4.306	110.842	581.157	5.24
6	(24.0, 104.5)	4.355	4.138	134.710	581.157	4.31
7	(32.0, 133.0)	4.158	3.950	180.684	581.157	3.22
8	(44.0, 174.9)	3.975	3.776	213.440	581.157	2.72
9	(52.0, 200.2)	3.850	3.658	236.808	581.157	2.45
10	(60.0, 223.5)	3.726	3.539	251.240	581.157	2.31

Note: k is the slope, k0.95 is 95% of the slope, *P_Q_* is the critical failure load, *P_m_* is the maximum load, and *P_m_*/*P_Q_* is the ratio of the maximum load to the critical failure load.

**Table 2:** Comparison of the two methods for calculating fracture toughness.

Specimen	a (mm)	B (mm)	W (mm)	*P_m_* (N)	*P_Q_* (N)		*K_Ic_* (KN·m^3/2^)	
					P_Q_^S^	P_Q_^A^	K_Ic_^S^	K_Ic_^A^
50% WF/Re-HDPE	13.90	30.01	30.01	581.15	236.81	558.80	1431.29	650.00
60% WF/Re-HDPE	14.10	29.85	29.85	651.31	332.92	632.65	1557.99	932.51
70% WF/Re-HDPE	13.80	30.12	30.12	766.81	582.15	744.84	1907.81	1581.94

Note: The superscripts S and A refer to calculations via the standard method and AE techniques, respectively.

**Table 3:** The fracture toughness of high-filled WPCs with different wood fiber contents.

Specimen	*P_Q_* (N)	*P_m_* (N)	*P_m_*/*P_Q_*	*K_Ic_* (MN·m^3/2)^	Variable Coefficient (%)
50% WF/Re-HDPE	558.798	581.15	1.05	1.431 ± 0.044	3.044
60% WF/Re-HDPE	632.649	651.31	1.071	1.558 ± 0.052	3.362
70% WF/Re-HDPE	744.842	766.813	1.029	1.908 ± 0.039	2.042
